# Multisystem Inflammatory Syndrome and Autoimmune Diseases Following COVID-19: Molecular Mechanisms and Therapeutic Opportunities 

**DOI:** 10.3389/fmolb.2022.804109

**Published:** 2022-04-14

**Authors:** Parastoo Hosseini, Mohammad Sadegh Fallahi, Gisou Erabi, Majid Pakdin, Seyed Mahdi Zarezadeh, Arezoo Faridzadeh, Sarina Entezari, Arina Ansari, Mohadeseh Poudineh, Niloofar Deravi

**Affiliations:** ^1^ Department of Virology, School of Public Health, Tehran University of Medical Sciences, Tehran, Iran; ^2^ Research Center for Clinical Virology, Tehran University of Medical Sciences, Tehran, Iran; ^3^ School of Medicine, Tehran University of Medical Sciences, Tehran, Iran; ^4^ Student Research Committee, School of Medicine, Urmia University of Medical Sciences, Urmia, Iran; ^5^ Student Research Committee, School of Medicine, Shiraz University of Medical Sciences, Shiraz, Iran; ^6^ Department of Immunology and Allergy, School of Medicine, Mashhad University of Medical Sciences, Mashhad, Iran; ^7^ Immunology Research Center, Mashhad University of Medical Sciences, Mashhad, Iran; ^8^ Student Research Committee, School of Allied Medicine, Shahid Beheshti University of Medical Sciences, Tehran, Iran; ^9^ Student Research Committee, School of Medicine, North Khorasan University of Medical Sciences, Bojnurd, Iran; ^10^ School of Medicine, Zanjan University of Medical Sciences, Zanjan, Iran; ^11^ Student Research Committee, School of Medicine, Shahid Beheshti University of Medical Sciences, Tehran, Iran

**Keywords:** autoimmune disease, COVID-19, SARS-CoV2, multisystem inflammatory syndrome, multisystem inflammatory syndrome in children (MIS-C)

## Abstract

Coronavirus disease 2019 (COVID-19), caused by severe acute respiratory syndrome-coronavirus 2 (SARS-CoV-2), has led to huge concern worldwide. Some SARS-CoV-2 infected patients may experience post–COVID-19 complications such as multisystem inflammatory syndrome, defined by symptoms including fever and elevated inflammatory markers (such as elevation of C reactive protein (CRP), erythrocyte sedimentation rate, fibrinogen, procalcitonin test, D-dimer, ferritin, lactate dehydrogenase or IL-6, presence of neutrophilia, lymphopenia, decreased albumin, and multiple organ dysfunction). Post–COVID-19 complications may also manifest as autoimmune diseases such as Guillain-Barré syndrome and systemic lupus erythematosus. Signaling disorders, increased inflammatory cytokines secretion, corticosteroid use to treat COVID-19 patients, or impaired immune responses are suggested causes of autoimmune diseases in these patients. In this review, we discuss the molecular and pathophysiological mechanisms and therapeutic opportunities for multisystem inflammatory syndrome and autoimmune diseases following SARS-CoV-2 infection with the aim to provide a clear view for health care providers and researchers.

## Introduction

Coronavirus disease 2019 (COVID-19) is caused by severe acute respiratory syndrome coronavirus 2 (SARS-CoV-2) virus, which was first reported in the city of Wuhan, China. The virus spread globally, and at the time of writing, more than 410 million cases and about 5 million deaths have been reported (Dong et al., 2020). Analysis of SARS-CoV-2 in terms of structural biology has categorized it into ORF6, 7a, 8, ORF3, E, M, ORF1ab, S, and ORF10. The spike protein enables the virus to adhere to the host cell membrane and the nucleocapsid protein (N protein) holds the RNA genome of the virus. The membrane and envelope along with spike protein constitute a viral envelope (Rota et al., 2003). The nonstructural RNA genome of ORF6, 7a, 8, ORF3, ORF1ab, and ORF10 consist of conserved information for replication of the genome to a great extent (Andersen et al., 2020).

Although SARS-CoV-2 is primarily known to cause substantial pulmonary disease, studies indicate that it can affect multiple organs and cause gastrointestinal, hepatic, cardiovascular, renal, neurological, ophthalmic, and cutaneous manifestations of COVID-19 (Johnson et al., 2020). Some patients experience post-acute COVID-19, extending beyond 3 weeks from the onset of the first symptoms, and chronic COVID-19, extending beyond 12 weeks (Greenhalgh et al., 2020). Post-acute COVID-19 is associated with complications such as dyspnea, decreased exercise capacity, thromboembolic events, palpitations, chest pain, fatigue, anxiety, depression, resolution of acute kidney injury (AKI), prolonged viral fecal shedding, hair loss, and multisystem inflammatory syndrome (MIS) both in children and adults (Nalbandian et al., 2021). MIS is defined by symptoms including fever and elevated inflammatory markers (more than two of the following: elevation of C reactive protein (CRP), erythrocyte sedimentation rate, fibrinogen, procalcitonin test, D-dimer, ferritin, lactate dehydrogenase or IL-6, presence of neutrophilia, lymphopenia, decreased albumin, multiple organ dysfunction, and exclusion of other plausible diagnoses) (Rodríguez et al., 2020; Tenforde and Morris, 2021).

SARS-CoV-2 infection may alter the immunologic system, resulting in states ranging from abnormal cytokine or chemokine production and maladaptive immune response to an increased number of activated macrophages, monocytes, and neutrophils and hyperactivated T cells (Rodríguez et al., 2020). Several studies reported autoimmune diseases such as immune thrombocytopenic purpura (ITP), Guillian-Barrė syndrome (GBS), Graves’ disease, systemic lupus erythematosus (SLE), antiphospholipid antibodies, and thrombosis associated with COVID-19 (Ehrenfeld et al., 2020; Rodríguez et al., 2020); however, MIS and autoimmune diseases can occur both para- and post-COVID-19 (Wang et al., 2015; Santos et al., 2021). Although the mechanism is unclear, some factors such as pro-inflammatory cytokines and chemokines, damage-associated molecular patterns (DAMPs), molecular mimicry, cross-reactive antibodies, and auto-antibodies may attribute to post-COVID-19 autoimmune diseases (Liu et al., 2021).

This review aims to provide a clear view for health care providers and researchers regarding the molecular and pathophysiological mechanisms and therapeutic opportunities.

### Post COVID-19 Multisystem Inflammatory Syndrome in Children (MIS-C)

MIS-C is an uncommon but serious medical condition which is associated with SARS-CoV-2 (Hasan et al., 2021). The disease seems to develop from a dysregulated immune response, resulting in endothelial dysfunction and a hyperinflammatory state, which eventually causes capillary leak, followed by multiorgan failure (Panaro and Cattalini, 2021). MIS-C is characterized by inflammation in multiple organs such as the brain, kidneys, heart, eyes, lungs, skin, or gastrointestinal system (Hasan et al., 2021). Therefore, the clinical picture can be wide, depending on the severity and organ manifestation (Panaro and Cattalini, 2021). According to a recent systematic review, the most common symptoms were gastrointestinal symptoms (71%), with the incidence of vomiting (25%), diarrhea (27%), and abdominal pain (36%), followed by mucocutaneous manifestations (conjunctivitis, strawberry tongue, skin rash, and dried-cracked lips). Fever, as a key criterion of MIS-C diagnosis, was present in all of the evaluated cases (Panaro and Cattalini, 2021; Radia et al., 2021). Furthermore, cardiac complications including acute myocardial injury, myocarditis, decreased ejection fraction, coronary artery aneurysm, pericarditis, valve dysfunction, pericardial effusion, arrhythmia, ventricular dilation, and tachycardia are reported (Panigrahy et al., 2020; Pouletty et al., 2020; Simpson and Newburger, 2020). Many gastrointestinal signs and symptoms of MIS-C overlap with those of acute appendicitis (Nakra et al., 2020; Belay et al., 2021; Martin et al., 2021).

Clinical criteria defined by the CDC (Center for Disease Control and Prevention) for MIS-C includes 21 years of age or less presenting with fever, laboratory findings in favor of inflammation, involvement of more than two organs; with no other differential diagnosis; and positive SARS-CoV-2 IgG or RT-PCR, definite exposure to SARS-CoV-2 within 4 weeks of symptom onset (Holstein, 2021). There is a 2- to 4-week delay in developing MIS-C after COVID-19 infection, and the peak of MIS-C lags behind the peak of acute SARS-CoV-2 infection (Feldstein et al., 2020; Verdoni et al., 2020). Serology tests have a higher probability of identifying MIS-C than reverse transcription polymerase chain reaction (RT-PCR), since MIS-C is a late form of the disease that occurs when the antibody rates are increasing, and the presence of the virus is no longer expected (Toubiana et al., 2020).

Laboratory findings have shown elevated levels of cytokines including IL-6, IL-8, IL-10, TNF-α, IFN-ɣ, sC5b-9 (associated with endothelial damage), IL-18, sIL-2R, CXCL9, elevated levels of acute-phase reactants including CRP and procalcitonin (PCT), and elevated vascular injury markers such as d-dimers and B-natriuretic proteins (Diorio et al., 2020; García-Salido et al., 2020). In addition, several factors are associated with cardiac injury, such as D-dimer, BNP, N-terminal -proBNP (NT-proBNP), and troponin-T are elevated in MIS-C patients (Diorio et al., 2020; García-Salido et al., 2020; Simon Junior et al., 2021). Also, increased levels of ferritin and erythrocyte sedimentation rate (ESR), lactic dehydrogenase and triglycerides, prolonged prothrombin time, lymphopenia, thrombocytopenia, neutrophilia, and hypoalbuminemia are seen (García-Salido et al., 2020; Lee et al., 2020; Panigrahy et al., 2020; Pouletty et al., 2020; Abdel-Haq et al., 2021; Simon Junior et al., 2021).

MIS-C shares some clinical features with Kawasaki disease (KD), which is a paediatric, self-limited, systemic inflammatory vasculitis, and macrophage activation syndrome (MAS); however, it is considered a different condition with a wide variety of clinical presentation (Al Maskari et al., 2021; Appleberry et al., 2021; Haoudar et al., 2021; Hasan et al., 2021; Jain et al., 2021; Tolunay et al., 2021). It is reported that 40% of patients with MIS-C met the criteria for either incomplete or complete KD (Feldstein et al., 2020). KD is typically known as a disease of young children < 5 years old, while MIS-C has been widely reported in a wide age range (from 1.6 to 20 years, with a median age of 6–11 years) (Abrams et al., 2020; Ouldali et al., 2020) In addition, the ethnicity of the MIS-C patient population is predominantly African-American/Hispanic while KD mostly affects the Asian population (Bukulmez, 2021). The pattern of coronary artery dilation in the two conditions is different from each other. The coronary involvement in MIS-C is usually mild and is detectable during the early phase of febrile illness, which rapidly resolves on short-term follow up in the majority of cases (Farooqi et al., 2021; Feldstein et al., 2021). On the other hand, the coronary artery dilation usually peaks after remission of the febrile illness in KD (McCrindle et al., 2017). The difference in severity and timing of coronary artery dilation in the two diseases can be justified with different pathologic mechanisms. It is assumed that coronary dilation in MIS-C is due to rising levels of circulating cytokines which is accompanied by endothelial cell dysfunction and probably edema resulting in mild dilation of the coronary arteries, and inflammatory cells infiltrate the coronary arteries in KD, resulting in disruption of elastin and collagen fibers and structural integrity loss, which eventually leads to aneurysms of the arteries (Orenstein et al., 2012). Autoantibody profiles have also been compared in patients with MIS-C and KD (Consiglio et al., 2020). The levels of antibodies to vascular endothelial cell proteins, like endoglin, were found to be higher than in healthy controls in both groups of patients; however, some autoantibodies (including that to discoidin I-like domain-containing protein 3 and EGF-like repeat) were overexpressed in KD compared with MIS-C. Moreover, plasma levels of endoglin were reported to be elevated in both groups of patients compared with healthy individuals, which raises the possibility that antibodies to endothelial cells might be the result, rather than the cause, of vascular damage. One other possibility is that the S protein superantigen of SARS-CoV-2 can cause aberrant activation of B cells (Bar-Meir et al., 2021).

MAS is characterized by unremitting fever, hepatic dysfunction, hyperferritinemia, pancytopenia, and coagulopathy, and it is commonly associated with systemic juvenile idiopathic arthritis (sJIA) and SLE (Crayne et al., 2019). Laboratory findings suggest that cytokine storm in MIS-C is relatively similar to that in MAS. However, MIS-C patients have higher fibrinogen, CRP, ESR, and prohormone-B-type natriuretic protein (proBNP) levels and lower ferritin levels and lymphocyte counts than MAS (Aydın et al., 2021; Otar Yener et al., 2021). In addition, MAS patients have higher IL-12, L-18, and CXCL9 than MIS-C patients (Lee et al., 2020). MIS-C patients have a significantly lower left ventricular ejection fraction, indicating a more severe disease than MAS (Aydın et al., 2021).

### Pathophysiology

#### Cytokine Storm

MIS-C patients show elevated levels of IL-6 (Kaushik et al., 2020; Lee et al., 2020; Evans and Davies, 2021). IL-6 functions through two signaling pathways termed classic-cis-signaling and trans-signaling. In classic-cis signaling, IL-6 binds to its transmembrane receptor (mIL-6R), which is expressed on hepatocytes, megakaryocytes, and several immune cells, resulting in dimerization of gp130, phosphorylation of STAT3, and also activation of Akt/mTOR and MAPK signaling pathways (Tanaka et al., 2014; Patra and Ray, 2021). In the trans-signaling pathway, IL-6 binds to soluble IL-6 receptor (sIL-6R), forming a complex that binds to gp130, which is ubiquitously expressed. Binding to gp130 activates the JAK/STAT3 pathway in cells lacking mIL-6R such as endothelial cells, vascular smooth muscle cells (VSMCs), and fibroblasts which further triggers the production of IL-6, IL-8, monocyte chemoattractant protein-1 (MCP-1), vascular endothelial growth factor (VEGF), and the reduction of E-cadherin expression on endothelial cells (Tanaka et al., 2016).

TNF-α is also increased in the acute phase of MIS-C (Carter et al., 2020). However, Consiglio et al. reported that TNF-α levels were significantly lower in patients with MIS-C than in adults with acute COVID-19 and TNF-α levels were relatively similar to those in healthy children (Consiglio et al., 2020). TNF-α is a proinflammatory cytokine. The binding of TNF-α to TNF-R1 induces recruitment of TRADD (TNF-R1-associated death domain protein), and TRADD further recruits FADD/MORT1, TRAF2, and death domain kinase RIP. FADD/MORT1 induces TNF-associated cell death, and RIP and TRAF2 are involved in the activation of NF-κB and JNK (Liu, 2005). NF-κB can induce several proinflammatory gene expressions and elevation of cytokines and chemokines and participates in inflammasome regulation (Liu, 2005).

SARS-CoV-2 infection can mediate the secretion of IL-6 and TNF-α *via* several mechanisms. For example, attachment of SARS-CoV-2 spike protein to Angiotensin-converting enzyme 2 (ACE2) receptors on respiratory epithelial cells and entry results in inflammatory cytokine production and a weak IFN response. Membrane-bound immunologic receptors and downstream signaling pathways mediate the proinflammatory response of pathogenic Th1 cells and intermediate CD14^+^CD16^+^ monocytes and subsequently cause cytokine storm by the infiltration of neutrophils and macrophages into the lung tissue (Hussman, 2020). Activated pathogenic Th1 cells release granulocyte-macrophage colony-stimulating factor (GM-CSF), which further stimulates CD14^+^CD16^+^ monocytes to secrete IL-6 and TNF-α (Zhou et al., 2020a).

In addition, SARS-CoV-2 viral genomic single-stranded RNA or other RNA compositions may act as pathogen-associated molecular patterns (PAMPs) and bind to pathogen recognition receptors (PRRs) such as TLRs and RLRs (Khanmohammadi and Rezaei, 2021). PAMP recognition leads to activation of IRF3/7 and NF-κB downstream signaling pathways resulting in the secretion of IFN-I and proinflammatory cytokines (Yang et al., 2021). Also, Hirano and Murakami indicated that activation of the NF-κB pathway leads to occupation and reduction of ACE2 surface receptors (Hirano and Murakami, 2020). Reduction of ACE2 expression results in an increase in angiotensin II, which binds to angiotensin receptor I and the complex through disintegrin and metalloprotease 17 (ADAM17) and induces TNF-α and sIL-6R production (Eguchi et al., 2018).

Kang et al. reported that IL-6 is positively correlated with plasminogen activator inhibitor-1 (PAI-1) and, through the trans-signaling pathway, can induce endothelial damage and coagulopathy in patients with COVID-19–related cytokine release syndrome (CRS) (Kang et al., 2020). Also, IL-6 can increase tissue factors on monocytes triggering the coagulation cascade and thrombin activation (Kang and Kishimoto, 2021). In addition, IL-6 is related to vascular damage through C5a expression and VE-cadherin disassembly (Kang and Kishimoto, 2021). MIS-C patients show elevated levels of IL-8 (Carter et al., 2020; Kaushik et al., 2020; Riollano-Cruz et al., 2021).

#### Cellular Immunity

Neutrophils play an essential role in the innate immune response. Carter et al. reported increased neutrophil CD64 median fluorescence intensity (MFI), a neutrophil activation marker, in the acute phase of MIS-C. Activated neutrophil levels are normalized in the resolution phase. Also, they reported decreased CD10 MFI on neutrophils, which implies decreased mature neutrophils (Carter et al., 2020). Neutrophils are capable of ferritin secretion, and elevated ferritin levels are seen in MIS-C patients (Simon Junior et al., 2021). Ferritin has an immunosuppressive and proinflammatory function. The immunosuppressive role includes suppressing the delayed type of hypersensitivity, suppressing antibody production, regulating granulomonocytopoiesis, and reducing phagocytosis by granulocytes through H-ferritin signaling pathways on lymphocytes, downregulation of CD2 and CXCR4, and inducing the production of IL-10 (Rosário et al., 2013). The proinflammatory role of ferritin is proposed by Ruddell et al., in which ferritin activated the TIM-2-independent pathway and further leads to the activation of NF-κB and production of proinflammatory cytokines such as IL-1β (Ruddell et al., 2009).

MIS-C patients have elevated levels of fibrinogen and D-dimer, indicating abnormal coagulopathy. Neutrophils can form neutrophil extracellular traps (NETs) that are associated with thrombosis and may play a role in MIS-C (Jiang et al., 2020a; Middleton et al., 2020). In the conventional NETosis pathway, activation of TLRs, receptors for IgG–Fc, complement, or cytokines lead to increased cytoplasmic calcium and elevated calcium levels activate protein kinase C (PKC) and phosphorylation of gp91phox (Kaplan and Radic, 2012). The phosphorylation of gp91phox results in the activation of phagocytic oxidase and production of reactive oxygen species (ROS) and rupture of granules and the nuclear envelope along with chromatin decondensation. NET release occurs after the rupture of the plasma membrane (Papayannopoulos et al., 2010). NETs can promote thrombosis through platelet and red blood cell adhesion and aggregation. DNA, histones, and proteases in NETs have procoagulant properties (Yang et al., 2016). NETs are also involved in morbid thrombotic events in patients with COVID-19 (Zuo et al., 2021). However, Seery et al. reported that NET production was similar in children with COVID-19 and healthy controls (Seery et al., 2021).

T cells may be involved in the pathogenesis of MIS-C. Consiglio et al. reported that in patients with MIS-C, total T cell frequencies were lower than in healthy controls and CD4^+^ distribution was similar between children with MIS-C and mild COVID-19. Central memory (CM), effector memory (EM), and terminally differentiated effector CD4^+^ T cells were higher and naïve CD4^+^ and follicular helper T cells were lower in MIS-C and COVID-19 patients than in children with KD. Compared to children with mild COVID-19, children with MIS-C had significantly lower CD4^−^ (mostly CD8^+^) T cells (Consiglio et al., 2020). Carter et al. observed decreased helper (CD4^+^), cytotoxic (CD8^+^), and γδ T cells in the acute phase of MIS-C. Levels of CD4+CCR7+ T cells (primarily naive T cells and a small proportion of CM T cells) were high and, during the acute phase, had higher HLA-DR MFIs, which is indicative of activation. Also, γδ T cells with antiviral properties were decreased in the acute phase (Carter et al., 2020).

Noval Rivas et al. proposed a superantigen hypothesis in which SARS-CoV-2 spike protein encodes a high-affinity superantigen-like sequence motif near the S1/S2 cleavage site of the spike protein that can bind to the T-cell receptor (TCR). This may result in excessive T-cell activation and proliferation due to its similarity to superantigenic Staphylococcal Enterotoxin B (SEB) (Noval Rivas et al., 2021).

#### Antibodies and Immune Complexes

Autoantibodies are involved in the pathogenesis of MIS-C. Consiglio et al. detected antibodies against endoglin in MIS-C patients. Endoglin is a glycoprotein expressed by endothelial cells, and it is crucial for structural integrity and is predominantly seen in the vascular endothelium and heart muscle. They reported that autoantibodies are possibly a consequence of tissue damage due to elevated plasma endoglin levels (Consiglio et al., 2020). Autoantibodies against the MAP2K2 and three members of the Casein kinase family (CSNK1A1, CSNK2A1, and CSNK1E1) are seen explicitly in MIS-C patients. The casein-kinase 2 pathway is involved in viral replication, and antibodies produced against the component of the mentioned pathway may attribute to the development of MIS-C (Consiglio et al., 2020).

#### Mechanism of Organ Damage

It has been proposed that the major mechanism of organ damage in individuals with MIS-C is antigen–antibody–mediated cytokine storm (Haslak et al., 2021). The underlying mechanism of myocardial injury in patients with MIS-C is not clearly understood. However, it has been assumed that acute viral myocarditis, systemic inflammation, hypoxia stress cardiomyopathy, and, less commonly, ischemia, which is the result of coronary involvement, may play a role in causing the damage (Sperotto et al., 2021). It is also not clear how exactly MIS-C is related to neurological involvement. It has been suggested that cellular edema of neurons, which is the subsequent result of immune-mediated neuronal damage and inflammatory response, may be the cause for neurological involvement in patients with MIS-C (Lin et al., 2021). Cross-reaction of the infectious agent with ocular-specific antigens is a proposed theory explaining the pathogenesis of uveitis in children with MIS-C (Wildner and Diedrichs-Möhring, 2020).

COVID-19 activates the thrombosis cascade through different mechanisms. It also leads to overexpression of PAI-1 (Ahmed et al., 2020). It is found that overexpression of PAI-1 is related to coronary artery aneurysm development in patients with KD (Senzaki et al., 2003), which can explain the risk of development of the condition on follow-up of patients with MIS-C (Patnaik et al., 2021).

### Treatment

The aim of the multidimensional approach toward MIS-C’s treatment is to modulate cytokine storm and inflammation. Treatment for fever, dehydration, stress ulcer prophylaxis, and hypercoagulability are considered the standard treatment choices (Beroukhim and Friedman, 2020). Table 1 summarizes the findings of studies on the potential treatments for MIS following COVID-19.

**TABLE 1:** Summary of studies on the therapeutic options for multi-system inflammatory syndrome following SARS-CoV2 infection.

Author	Year	Type of study	Patient(s)	Clinical manifestations of MIS	Intervention/drugs	Outcomes of treatment	References
Hasan et al	2021	Case series	6-male 2-female	Fever, rash, tachycardia, hypotension, abdominal pain, diarrhea, vomiting, decreased oral intake, cough, sore throat, conjunctivitis	IVIG, corticosteroids, antibiotics, anticoagulants, epinephrine/norepinephrine, aspirin, Interleukin-1ra inhibitor	Survived	Hasan et al. (2021)
Pouletty et al	2020	Cohort	8-male 8-female	Skin rash, hands and feet erythema/oedema, conjunctivitis, dry cracked lips, cervical lymphadenopathy	Intravenous immunoglobulin, steroids, Anti–IL-1 treatment, Anti–IL-6 treatment, hydroxychloroquine	Survived	Pouletty et al. (2020)
Appleberry et al	2021	Case report	Male	Fever, seizures	Steroids, IVIG, midazolam	Survived	Appleberry et al. (2021)
Diorio et al	2020	Cohort	2-male 4-female	Fever, rash, cracked lips, conjunctivitis, myocardial dysfunction, shock, requiring intubation and vasoactive, severe abdominal pain, diarrhea	IVIG, aspirin, steroids	Survived	Diorio et al. (2020)
Abdel-Haq et al	2021	Case series	15-male 18-female	Fever, vomiting, diarrhea, abdominal pain, respiratory distress, skin rash, neck tenderness, lymphadenopathy, chest pain, hypotension, cardiac involvement, coronaries dilated, ejection fraction	IVIG, infliximab	Survived	Abdel-Haq et al. (2021)
Lee PY et al	2020	Case series	16-male 12-female	Fever, conjunctivitis, hypotension/shock, skin rash, extremity swelling/erythema, acute kidney injury	IVIG, methylprednisolone, anakinra, remdesivir, antibiotics, aspirin, enoxaparin	Survived	Lee et al. (2020)
Riollano-Cruz et al	2021	Case series	11-male 4-female	Rash, conjunctivitis, swollen hands and feet, tachycardia, hypotension, abdominal pain, emesis, diarrhea, myalgia, chest pain, nausea, sore throat, headache, neck stiffness	Vancomycin, cefepime, metronidazole, ivig, tocilizumab, enoxaparin, clindamycin, norepinephrine, vasopressin, amiodarone, lidocaine, vancomycin, meropenem, anakinra, remdesivir, linezolid, cefepime, dobutamine	Survived	Riollano-Cruz et al. (2021)
Consiglio et al	2020	Case control	8-male 3-female	Encephalitis, headache, conjunctivitis, rash, swollen hands and feet, abdominal pain, myocarditis, sore throat, lymphadenopathy, vomiting, cough	L-1RA (anakinra), hydroxychloroquine, IVIG, steroids	Survived	Consiglio et al. (2020)
Guanà, Riccardo et al	2021	Case report	7-year-old boy	Low-grade fever, conjunctivitis, gastroenteritis-like symptoms, hyperpyrexia associated with asthenia, and anorexia	2 mg/kg/day intravenous methylprednisolone for 1 week, followed by 1.5 mg/kg/day oral prednisolone for an additional 1 week	Survived	Guanà et al. (2021)
Sweeny, Katherine F et al	2021	Case report	16-year-old boy	Abdominal pain, diarrhea, hematochezia, severe active gastro-duodenitis, patchy colitis	Steroids, intravenous immunoglobulin, and infliximab	Survived	Sweeny et al. (2021)
Esteve-Sole, Ana et al	2021	Cohort	18-female 19-male	Increased gastrointestinal and neurological symptoms, increased lymphopenia and thrombopenia, and decreased neutrophilia	Intravenous immunoglobulin [IVIG], steroids, tocilizumab, anakinra	Survived	Esteve-Sole et al. (2021)
Almoosa, Zainab A et al	2020	Case series	5-female 5-male	High grade fever, GI symptoms, diarrhea, abdominal pain and emesis, conjunctivitis, lymphadenopathy, irritability, shock	Ventilatory support, vasoactive support, IVIG, antibiotic, steroids, antiviral (favipiravir), heparin, aspirin	8 patients survived	Almoosa et al. (2020)
J. J. Rodriguez-Smith rt al	2021	Cohort	8-female 11-male	Lymphopenia, thrombocytopenia, marked elevation of inflammatory markers, hyperferritinaemia, elevated cardiac biomarkers, and acute renal injury	Intravenous immunoglobulin, corticosteroids, IL-1 receptor antagonist anakinra	Survived	Rodriguez-Smith et al. (2021)
Rojahn, Astrid Elisabeth et al	2020	Case report	Child - unknown sex and age	Piperacillin/tazobactam and intravenous fluids, vasopressor therapy (noradrenaline)	Abdominal pain, nausea, vomiting, frontal headache, and reduced general condition	Survived	Rojahn et al. (2020)
Karthika IK et al	2021	Case report	14-year-old girl	Headache, fever, bilateral uveitis, unilateral cervical lymphadenopathy, oral mucosal changes, and abdominal pain	Intravenous immunoglobulin (IVIG) and oral steroids	Survived	Karthika et al. (2021)
Shahein AR et al	2021	Case report	6-year-old boy	Phlegmonous ileocolitis, myocarditis, shortness of breath, fatigue, tachypnea	Intravenous ceftriaxone and metronidazole, immunoglobulin, and methylprednisolone	Survived	Shahein et al. (2021)
Garcia-Dominguez M et al	2020	Case series report	1 boy and 3 girls	Fever, gastrointestinal involvement, general malaise, asthenia, and adynamia	Vasoactive therapy, fluid resuscitation, and also, 3 of them received IVIG	Survived	Garcia-Dominguez et al. (2020)
Balasubramanian S et al	2020	Case report	8-year-old boy	Fever, respiratory symptoms	IVIG, tocilizumab, ceftriaxone, and azithromycin	Survived	Balasubramanian et al. (2020)
Whittaker et al	2020	Case series	58 children (20 female)	Fever, vomiting, abdominal pain, diarrhea, rash, conjunctival injection, inflammation, myocardial injury, shock, and coronary artery aneurysms	Inotropic support, IVIG, corticosteroid, anakinra, infliximab	57 patients survived	Whittaker et al. (2020)
Dufort et al	2020	Case report	95 patients (53 male)	Fever, chills, tachycardia, gastrointestinal symptoms, rash, conjunctival injection, mucosal changes	Vasopressor support, ICU, mechanical ventilation, IVIG, systemic glucocorticoids, vasopressor support, echocardiogram	94 patients survived	Dufort et al. (2020)
Godfred et al	2020	Case report	570 patients (254 female)	Fever, rash, conjunctivitis, peripheral edema, gastrointestinal symptoms, shock, and elevated inflammation markers, cardiac damage	IVIG, steroids, antiplatelet and anticoagulation and vasoactive medication, respiratory support, ventilation, dialysis, immune modulators	560 patients survived	Godfred-Cato et al. (2020)

#### Fluid Resuscitation and Antibiotic

It is vital to administer antibiotic and hydrate the patients who are septic and hypotensive during physical exam for MIS-C. Furthermore, it is sometimes necessary for the patients to receive inotropes until bacterial infection has been ruled out (Jiang et al., 2020a).

#### Intravenous Immune Globulin (IVIG)

IVIG is considered a first-line treatment in patients with KD and can reduce the risk of coronary artery lesion (CAL) (Newburger et al., 2004). The mechanism of IVIG is not fully understood. However, it has been suggested that IVIG is involved in the blockage of the Fc receptor, neutralization of pathogenic products, immune-modulation, regulation of T-cell activity, and cytokine production (Kuo et al., 2016). Therefore, the American Academy of Pediatrics and the American Heart Association guideline suggested the use of high doses of immunoglobulins (2 g/kg) within 8–12 h with high doses of aspirin for the treatment of KD (Newburger et al., 2004). Several studies reported the use of IVIG for the treatment of MIS-C either alone or in combination with other therapies (Consiglio et al., 2020; Diorio et al., 2020; Garcia-Dominguez et al., 2020; Kaushik et al., 2020; Appleberry et al., 2021; Hasan et al., 2021; Jonat et al., 2021; Karthika et al., 2021; Shahein et al., 2021; Yasuhara et al., 2021). For example, in a study by Pouletty et al. on 16 patients with MIS-C, 15 patients received IVIG, and only 5 (31%) showed remission after single IVIG treatment and others required second-line treatment (Pouletty et al., 2020).

#### IL-6 Inhibitors

Tocilizumab is a recombinant humanized monoclonal IgG1κ antibody and binds to IL-6R and inhibits cis-signaling, trans-signaling, and trans-presentation by preventing IL-6 attachment. Tocilizumab has been approved for the treatment of rheumatoid arthritis and systemic juvenile idiopathic arthritis (Pelaia et al., 2021). In addition, a systematic review and meta-analysis by Wei et al. on 26 studies showed that tocilizumab is associated with a lower risk of mortality and the need for mechanical ventilation in COVID-19 patients (Wei et al., 2021).

In a case report of a child with hyperinflammatory syndrome and COVID-19, tocilizumab (8 mg/kg IV over 2 h) was used 72 h after IVIG infusion, resulting in settled fever spikes and reduction of inflammatory parameters to normal (Balasubramanian et al., 2020a).

#### IL-1 Inhibitors

IL-1 may play a significant role in the MIS-C pathology. IL-1α and IL-1β are the two cytokines which mediate inflammatory response to lung injury. Inflammation is caused by the production of IL-1α by injured epithelium and endothelial tissues, while IL-1β is released by invading myeloid cells. The IL-1 receptor antagonist (IL-1Ra) is the main mechanism that prevents excessive inflammation caused by either cytokine. IL-1Ra inhibits the receptor that transmits the pro-inflammatory effects of both IL-1α and IL-1β. Anakinra is the recombinant form of the naturally occurring IL-1 receptor antagonist (IL-Ra), which prevents the binding of IL-1α and IL-1β to IL-1R (Cavalli and Dinarello, 2018; Pasin et al., 2021). A meta-analysis by Pasin et al. involving a total of 184 COVID-19 patients showed that anakinra was associated with decreased mortality rate and requirement of mechanical ventilation (Pasin et al., 2021). Lee et al. used anakinra (doses ranging from 5 to 13 mg/kg/day) in five patients with MIS-C and reported that adding anakinra was associated with improvement of the inflammatory process in patients. Clinical improvement was seen in all cases, with resolution of fever, cessation of inotrope treatment, and improvement of inflammatory markers. CRP, d-dimer, and ferritin were also decreased (Lee et al., 2020).

#### TNF Inhibitors

Infliximab is a recombinant DNA-derived chimeric human-mouse IgG monoclonal antibody, which binds to the soluble and membrane form of TNF and blocks TNF signaling and biological activities (Guo et al., 2013). Administration of infliximab was associated with the reduction of inflammatory markers and cytokine concentrations in patients with COVID-19 (Robinson et al., 2020).

Dolinger et al. reported a child with Crohn’s disease, MIS-C, and COVID-19 treated with infliximab (10 mg/kg). The treatment resulted in resolved fever, tachycardia, and hypotension within hours. Also, IL-6 and IL-8 concentrations decreased with TNF-α normalization (Dolinger et al., 2020).

Abdel-Haq et al. used high-dose infliximab (10 mg/kg) as the second-line treatment in 12/13 patients with MIS-C. The results showed that infliximab was associated with the resolution of fever, improvement of cardiac function, and improvement of coronary artery dilatation (Abdel-Haq et al., 2021).

#### Corticosteroids

Various studies reported using corticosteroids, including methylprednisolone, prednisolone, hydrocortisone, and dexamethasone for the treatment of MIS-C (Godfred-Cato et al., 2020; Jiang et al., 2020a; Cheung et al., 2020; Diorio et al., 2020; Lee et al., 2020; Panigrahy et al., 2020; Pouletty et al., 2020; Zou et al., 2021).

#### Anticoagulation

Low dose of aspirin (3–5 mg/kg/day; maximum 81 mg/day) should be used in MIS-C patients and continued until the platelet count is normalized and normal coronary arteries are confirmed at ≥4 weeks subsequent to diagnosis. The treatment should be avoided in patients with active bleeding, significant bleeding risk, and/or a platelet count of ≤80,000/μl. It is also highly recommended to prescribe enoxaparin for patients who suffer from coronary artery aneurysms with a z-score of ten or higher, patients with an ejection fraction less than 35%, and patients with a documented thrombosis. In other patients, decisions about prescribing anticoagulation should be made based on the individual risk factors of patients (Henderson et al., 2021).

#### Supportive Care

Patients with MIS-C who rapidly deteriorate need more intensive care. Extra corporeal membrane oxygenation is such a piece of equipment which may need to be used (Belhadjer et al., 2020).

**FIGURE 1:**
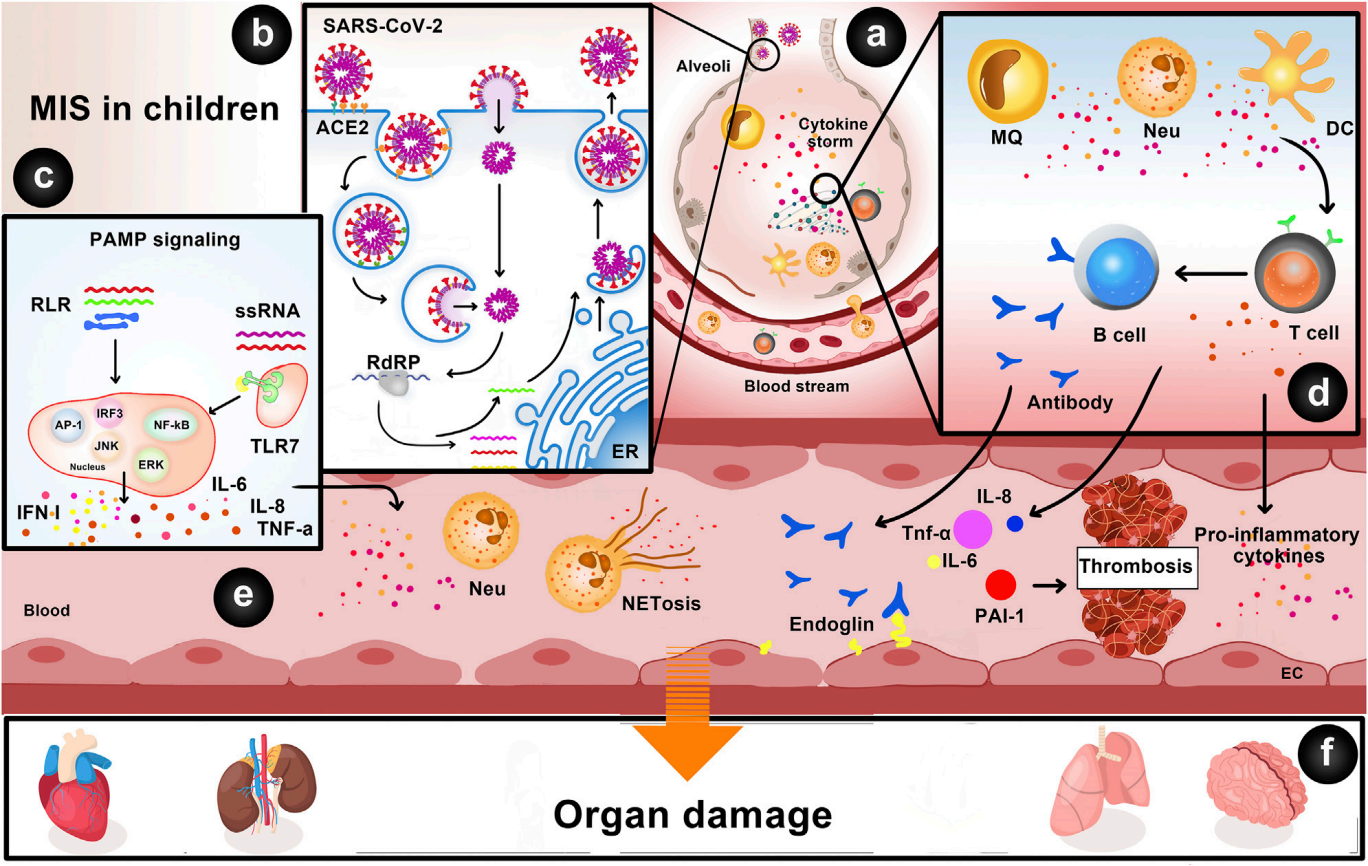
Underlying mechanisms of multisystem inflammatory syndrome in children. **(A,B)** The binding of SARS-CoV-2 spike protein to ACE2 receptors on respiratory epithelial cells leads to its cell entry and viral genome replication via RNA-dependent RNA polymerase (RdRP). Membrane-bound immunologic receptors and downstream signaling pathways mediate the pro-inflammatory response resulting in cytokine storm through the infiltration of neutrophils and macrophages into the lung tissue. **(C)** SARS-CoV-2 viral genomic ssRNA or other RNA compositions may act as PAMPs and bind to TLRs and RLRs, leading to the activation of IRF3, NF-κB, MAPK/ERK, AP-1, and JNK signaling pathways and secretion of IL-6, IL-8, TNF-α, and IFN-I. **(D)** Cytokines produced by macrophages, neutrophils, and dendritic cells cause T cell–dependent activation of B cells and production of antibodies and autoantibodies (e.g., anti-endoglin). **(E)** Neutrophils can form NETs, which are released subsequent to the rupture of the plasma membrane. NETs, IL-6, IL-8, TNF-α, and PAI-1 induce endothelial damage and thrombosis. **(F)** Cytokine storm, autoantibodies, and activation of immune cells lead to multiple organ damage, including the lungs, brain (e.g., cellular edema of neurons), heart (e.g., acute viral myocarditis), and kidneys (e.g., acute kidney injury).

Figure 1 summarizes the underlying mechanisms of post COVID-19 MIS-C.

### Post COVID-19 Multisystem Inflammatory Syndrome in Adults (MIS-A)

MIS can occur at any age and can arise synchronously with SARS-CoV-2 infection or as a postinfectious phenomenon (Dabas et al., 2021). MIS is a new disease related with SARS-CoV-2 that has also been seen in adults (Mieczkowska et al., 2021). According to CDC, MIS‐A is defined by the following criteria:

(1) severe illness requiring hospitalization in a person aged ≥21 years;
(2) a positive test result for current or previous SARS‐CoV‐2 infection at admission or during the previous 12 weeks;
(3) severe dysfunction of one or more extrapulmonary organ systems;
(4) laboratory evidence of severe inflammation; and
(5) absence of respiratory illness.

### Molecular Mechanisms

From a pathophysiological perspective, virus-infected cells undergo pyroptosis. This process involves the release of both cellular and viral components collectively called DAMPs and PAMPs (Freeman and Swartz, 2020; Shenoy, 2020). In the context of acute illness, DAMPs and PAMPs are recognized by the components of the innate immune system and the antigen-presenting cells (APCs) resulting in the release of interleukin (IL)-1β from the activated neutrophils and macrophages (Leyfman et al., 2020). IL-1β initiates local inflammation, which further stimulates the recruitment and activation of neutrophils, lymphocytes, and macrophages that release cytokines such as IL-6, interferon-γ, inducible protein-10, and monocyte chemoattractant protein-1 (MCP-1) (Leyfman et al., 2020). Pulmonary tissue damage secondary to SARS-CoV-2 pneumonia also contributes to asymptomatic or minimally symptomatic hypoxemia (Galwankar et al., 2020), which itself is a potent inducer of IL-6 and other cytokines (Leyfman et al., 2020). All these processes combine to produce a CRS potentiating the inflammatory damage in the lungs, kidneys, heart, brain, and gastrointestinal tract and leading to AMIS-COVID-19 (Leyfman et al., 2020). Nuclear factor-kappa B (NF-κB) plays an important role in cellular synthesis and development of CRS (Hirano and Murakami, 2020). Inactivation of NF-κB has been shown to effectively dampen CRS and prevent the development of AMIS-COVID-19 (DeDiego et al., 2014). The first step in the COVID-19–related inflammatory cascade is IL-1β production that is initiated upon recognition of PAMPs and DAMPs by a multiprotein cytosolic complex called the inflammasome. The inflammasome activation is known to be inhibited by colchicine, an agent used to treat acute attacks of gout and familial Mediterranean fever (Molad, 2002; Leung et al., 2015).

Although the exact mechanism of MIS-A is unknown, it appears by reason of a delay in the cytokine storm associated with the initial infection (Parpas et al., 2021). Viral infections are caused by a mechanism considered antibody-dependent enhancement (ADE), which increases the level of neutralizing antibodies and may lead to pathogenicity, while SARS-CoV-2 antibodies are supposed to be have protective and neutralizing properties. The exact cause of MIS-A is not known, but it may be due to a malfunction of the innate and adaptive host immune system that causes multi-organ failure, or it may be by reason of homology between SARS-CoV-2 spike protein and staph enterotoxin B super-antigen structure and sequence resulting in a hyper-inflammatory state (Al-Falahi et al., 2021).

### Treatment

Until now, there have been no widely accepted guidelines for the ideal therapeutic approach to adults with MIS. According to pathophysiological and clinical resemblance between MIS and incomplete KD and the effectiveness of IVIG in TSS, the literature indicates that MIS similar to KD should be cured with immune-modifying agents, first-line glucocorticoids, and IVIG to invert the inflammatory response. Moreover, current reports indicate that the combination of IVIG and steroid therapy may have better outcomes for treatment than IVIG monotherapy in KD (Henderson et al., 2020). Consequently, the value of glucocorticoids as first-line treatment for hyperinflammatory syndromes remains incontestable, despite the role of biologicals remaining unclear so far. Despite the fact that the exact efficiency in the long term remains unclear, patients in limited case series demonstrate hopeful results (Godfred-Cato et al., 2020; Jiang et al., 2020; Dufort et al., 2020; Henderson et al., 2020; Riphagen et al., 2020; Verdoni et al., 2020; Whittaker et al., 2020). The suggested treatment guideline for patients identified by MIS related to SARS-CoV-2, according to The American College of Rheumatology, consists of the following.

#### IVIG

The suggested dose of IVIG for patients with KD-like features is similar to what is used for KD, 2 g/kg (up to 70–80 g) body weight over a period of at least 8–12 h. In patients who have no or poor response, the administration of a second dose of IVIG can be regarded (Henderson et al., 2020).

#### Glucocorticoids

Severe cases with cardiac involvement, TSS, or hemophagocytic lymphohistiocytosis (HLH)-like course of the disease should be treated with a combination of IVIG and high doses of glucocorticoids. For the suggested dose of glucocorticoids, methylprednisolone during the early life-threatening stage in a regimen of 1 mg/kg body weight daily, or in more severe cases based on clinical characteristics and laboratory findings, methylprednisolone 30 mg/kg pulse therapy once daily during 1–3 days, and in cases with secondary HLH or central nerve system involvement, dexamethasone 10 mg/m2 once daily seems to be helpful. When the disease has reached the final stages and the patient is going to be dismissed from hospital, the oral dose of prednisolone can be decreased over a period of weeks to minimize the risk of relapse. The supporting evidence for using immune-modifying therapy is from previous case series, describing similar patient populations in the same health conditions, like KD, HLH, and TSS. In these case series, 75% of the cases were treated alike with IVIG, and they demonstrated clinical and cardiac recovery after treatment (Godfred-Cato et al., 2020; Dufort et al., 2020; Riphagen et al., 2020; Verdoni et al., 2020; Whittaker et al., 2020). In other limited case series, about 55% of the patients were treated with glucocorticoids in different doses. Before administrating IVIG in these patients, it is essential to obtain blood for blood cultures in analysis of possible pathogens and serologic SARS-CoV-2 test.

#### Biologicals

The biological anakinra (interleukin-1 receptor antagonist) is advised for patients with uncontrolled growing disease activity, severe secondary HLH, or shock by cardiac involvement in spite of the started therapeutic process according to steps 1 and 2—suitable because of its safety profile and short half-life.

Downing, S., et al. (2020) reported a case of successful combined pharmacotherapy for a patient with MIS-A and COVID-19 using colchicine, aspirin, and montelukast. The studied patient indicated remarkable recovery within 24 h of the starting of a colchicine-based regimen. Aspirin is the second in the suggested regimen that inhibits COX1&2 irreversibly and acts as an anti-inflammatory drug. The antiplatelet action of aspirin is mediated through the deterrence of TXA2 generation. Aspirin also has demonstrated antiviral effects against RNA viruses of the respiratory tract including influenza A viruses and rhinoviruses by its action on the regulation of the NF-κB pathway (Kopp and Ghosh, 1994; Yin et al., 1998; Glatthaar-Saalmüller et al., 2017). The third agent is montelukast, a cysteinyl leukotriene 1 receptor antagonist that is used to decrease bronchial inflammation in asthma (Pizzichini et al., 1999). Montelukast can also regulate the generation of IL-6, TNF-α, and MCP-1 through deterrence of NF-κB (Maeba et al., 2005). Montelukast may also have a direct antiviral effect on the SARS-CoV-2 main protease enzyme. Computer modeling studies propose that montelukast should have high-affinity binding to the active pocket of the main protease enzyme (Wu et al., 2020). Consequently, montelukast may have a bimodal action as a leukotriene antagonist and a protease inhibitor (Downing et al., 2020).

### Autoimmune Diseases Following COVID-19 Infection

Autoimmune diseases constitute a wide range of diseases characterized by the disruption of tolerance to self-antigens resulting in pathological changes and disruption of the target tissue’s function. Both genetic and environmental factors can trigger autoimmune diseases. Environmental factors include nutrition, microbiota, infections, xenobiotics, pharmaceutical agents, hormones, ultraviolet light, silica solvents, collagen or silicone implants, heavy metals, and vaccines (Wang et al., 2015). In addition, viruses are a major environmental factor associated with autoimmune diseases such as autoimmune hepatitis (Epstein–Barr virus), autoimmune myocarditis (Coxsackie virus), GBS (Zika virus), and multiple sclerosis (Epstein–Barr virus, Theiler’s virus, Varicella-zoster virus, Measles virus, and Cytomegalovirus) (Smatti et al., 2019).

It has been suggested that SARS-CoV-2 can trigger autoimmune diseases. In a systematic review of 64 articles, Shaikh et al. concluded that GBS is recognized as one of the presentations of the COVID-19 disease (Sheikh et al., 2021). In a systematic review by Saad et al. about 33 patients with autoimmune diseases after COVID-19, there were sixteen cases of GBS, eight cases of autoimmune hemolytic anemia, three cases of ITP, two cases of KD, and one case of subacute thyroiditis. They concluded that COVID-19 is involved in the development of various autoimmune diseases (Saad et al., 2021). In a review of 57 patients, Alonso-Beato et al. reported that ITP could occur both in mild and severe COVID-19 and during the course of the disease, and patients showed higher bleeding rates than in other ITP series (Alonso-Beato et al., 2021). In a prospective cohort study, Garjani et al. reported that in 57% of the patients (230 of 404 patients), COVID-19 infection leads to exacerbation of multiple sclerosis (MS) symptoms (Garjani et al., 2021). Also, several studies reported SLE following COVID-19 infection (Bonometti et al., 2020; Slimani et al., 2021; Zamani et al., 2021; Gracia-Ramos and Saavedra-Salinas, 2021).

The mechanism of post-COVID-19 autoimmunity is unclear; however, several factors may attribute to the condition. The third phase of COVID-19 infection is associated with acute respiratory failure, shock, immunothrombosis, multiorgan dysfunction or failure, and death (Berlin et al., 2020). The immune response in the third phase is characterized by hyperinflammation and cytokine storm (Wilson et al., 2020). The hyperinflammation mechanism can be associated with defects in type 1 and 3 IFN and dysregulated innate and adaptive immunity and can be stimulated by pathogen-driven factors and damage-associated markers (Winchester et al., 2021). A recent study by *Pan* et al. showed that SARS-CoV-2 N protein (involved in virus replication, assembly, and immune regulation) could interact with NLRP3 inflammasome resulting in cytokine storm and lung damage in mice (Pan et al., 2021).

Autoantibodies can be involved in the autoimmunity following COVID-19 infection. Several studies reported the presence of autoantibodies such as antinuclear antibodies (ANA), anti-SSA/Ro antibodies, and anti-IFN-I antibodies (Bastard et al., 2020; Zhou et al., 2020b). Antiphospholipid antibodies such as lupus anticoagulant, anti-cardiolipin, and anti-β2-glycoprotein I are correlated with immunocoagulopathy and thrombosis. They are detected in several cases of COVID-19 (Abdel-Wahab et al., 2016; Harzallah et al., 2020). Zuo et al. suggested that higher levels of antiphospholipid antibodies are related to the release of NETs, higher platelet counts, and more severe respiratory symptoms. They also reported that IgG extracted from patients with the antiphospholipid syndrome (APS) can initiate NET release from isolated neutrophils, and injection of the IgG to mouse models promotes venous thrombosis (Zuo et al., 2020). Table 2 summarizes the potential molecular mechanisms of and therapeutic options for post-COVID-19 autoimmune diseases.

**TABLE 2:** Summary of molecular mechanisms of and therapeutic options for autoimmune diseases following SARS-CoV2 infection/vaccination.

Diseases	Molecular mechanisms	Therapeutic option	References
Mucous membrane pemphigoid (MMP)	• Subepidermal autoimmune blistering disease	• Methylprednisolone	Drenovska et al. (2021)
	• Oral-pharyngeal erosions	• Dapsone	
	• Severe ocular scarring	• Azathioprine	
Autoimmune bullous dermatoses (AIBD): Pemphigus vulgaris (PV)	• Autoantibodies (desmoglein 1 and desmoglein 3)	• Systemic corticosteroids	Drenovska et al. (2021)
	• Painful mucosal and cutaneous erosions	• Antibiotics	
	• Flaccid bullae	• Acyclovir	
		• IVIG	
Autoimmune bullous dermatoses (AIBD): Bullous pemphigoid (BP)	• Subepidermal autoimmune blistering disease	• Topical and systemic corticosteroids	Drenovska et al. (2021)
	• Pruritus and blister formation on an erythematous base	• IVIG	
		• Deoxycycline and dapsone	
Long COVID: an estrogen-associated autoimmune disease	• Autoantibodies	• Personalized medicine based on the sex and appearance of autoantibodies	Ortona et al. (2021)
	• Sex hormones		
Myasthenia gravis	• Autoantibodies against acetylcholine receptor	• Pyridostigmine bromide	Restivo et al., 2020; Sriwastava et al. (2021b)
	• Increased inflammatory markers: interleukin-6, CRP, ferritin, fibrinogen, D-dimer	• Prednisone	
	• MG composite score = 3	• IVIG	
Hashimoto’s thyroiditis	• Increased TSH and thyroid peroxidase antibody levels	• Levothyroxine	Tee et al. (2021)
	• Low free thyroxine		
Systemic lupus erythematosus (SLE)	• Elevation of LDH, CRP, and ferritin	• Methylprednisolone	Bonometti et al. (2020); Slimani et al., 2020; Zamani et al. (2021)
	• Proteinuria	• Hydroxychloroquine	
	• Thrombocytopenia	• Cyclophosphamide	
	• Motor and sensory polyneuropathies	• Gabapentin	
	• Pleural effusion	• Vitamin B	
	• Low complement		
	• Increased anti-La/SSB, anti-SSA/Ro, anti-cyclic citrullinated peptides (anti-CCP) and anti-double-stranded deoxyribonucleic acid antibody (anti-dsDNA) antibodies, anticardiolipin immunoglobulin G (IgG) and immunoglobulin M (IgM) antibodies, anti‐β2‐glycoprotein I IgG, and immunoglobulin A		
	• (IgA) antibodies		
	• Lupus nephritis class I		
Varicella‐like rash	• Mild thrombocytopenia	• No specific treatment	Genovese et al. (2020); Slimani et al. (2020)
	• Pathophysiological mechanism remains unknown		
Graves’ disease	• Suppressed TSH	• Thiamazole	Mateu-Salat et al. (2020)
	• Free thyroxine (normal/increased)	• Propranolol	
	• Elevated free triiodothyronine		
	• + TSH receptor antibodies		
	• + Thyroperoxidase and thyroglobulin antibodies		
	• Increased thyroid iodine uptake		
Generalized pustular psoriasis	• Mild hypocalcemia	• Oral acitretin	Shahidi Dadras et al. (2021)
	• Neutrophilia	• Tapered oral prednisolone	
	• Elevation of creatinine level		
Autoimmune disease following Covid-19 vaccination			
Autoimmune hepatitis	• Biopsy consistent with autoimmune hepatitis	• Prednisolone	Bril, (2021)
	• ALT/AST increasing in blood		

TSH = thyroid-stimulating hormone, RBC= red blood cell, LDH= Lactate Dehydrogenase, MG= myasthenia gravis, ALT= alanine aminotransferase, AST= aspartate aminotransferase

### Similarities and Differences Between MIS-C and Autoimmune Diseases

Autoantibodies are involved in the pathogenesis of various autoimmune diseases such as Graves’ disease, myasthenia gravis, and SLE (Ludwig et al., 2017). As mentioned earlier, MIS-C patients show elevated levels of autoantibodies against endoglin, MAP2K2, and three Casein kinase family members, including CSNK1A1, CSNK2A1, and CSNK1E1 (Consiglio et al., 2020).

Moreover, a recent study identified 189 and 108 peptide candidates for IgG and IgA autoantigens, respectively. Some of the significant autoantibodies in MIS-C patients were anti-La (also seen in SLE and Sjogren’s disease) and anti-Jo-1 (also seen in idiopathic inflammatory myopathies) (Gruber et al., 2020). Also, Porritt et al. identified eight autoantigens in MIS-C that were previously seen as autoantigens in autoimmune diseases, including TROVE2 (SLE and Sjogren’s syndrome), KLHL12 (primary biliary cirrhosis and Sjogren’s syndrome), HK1 (primary biliary cirrhosis), ATP4A (type I diabetes and corpus atrophic gastritis), and FAM84A (inflammatory bowel disease) (Porritt et al., 2021). Bastard et al. reported that at least 10% of the patients with severe COVID-19 have autoantibodies against type I IFNs. Neutralizing autoantibodies against type I IFNs are also seen in patients of autoimmune polyendocrinopathy syndrome type I and SLE and may attribute to the pathogenesis of MIS-C (Bastard et al., 2020). However, several autoantigens in MIS-C were not associated with autoimmune diseases, and some had a tissue-specific expression, including endothelial and cardiac tissue (P2RX4, ECE1, and MMP14) and gastrointestinal tract (MUC15, TSPAN13, and SH3BP1) (Gruber et al., 2020).

The immune cell profiles of MIS-C patients show characteristics like autoimmune diseases. For example, Expansion CD11c + B cells expressing TBX1, along with decreased expression of CXCR5, CD21, CD24, and CD38, are seen in patients with MIS-C and SLE (Hoste et al., 2022). Also, Porritt et al. reported a high expression of IGHV4-39, a gene associated with autoreactive B cells, in an RNA cluster of MIS-C patients. IGHV4-39 expression was previously seen in autoreactive B cells of MS patients (Porritt et al., 2021). Furthermore, Ki67 + CD4^+^ T cells with high expression of ICOS*,* PDCD1, MAF, and IL-21 and low CXCR5 expression are seen in MIS-C and rheumatoid arthritis (Rao et al., 2017; Ramaswamy et al., 2021). Neutrophils are involved in various autoimmune diseases such as MS, SLE, inflammatory bowel disease, rheumatoid arthritis, and type I diabetes (Wang et al., 2018). Similarly, in MIS-C patients, FcγR and complement pathways of neutrophil activation are seen along with pathogenetic mechanisms such as NETosis, tissue damage caused by ROS and protease production, and cytokine and chemokine expression (Porritt et al., 2021).

Activation of complement system proteins by autoantibodies can result in leukocyte activation, cytotoxicity, and tissue damage, and they play a significant role in the pathogenesis of autoimmune diseases (Thurman and Yapa, 2019). Severe MIS-C patients show high expression of C1qA, C1qB, and C1qC involved in classical complement activation mediated by the abundance of autoantibody immune complexes (Porritt et al., 2021). However, in SLE, C1q deficiency is considered a risk factor that leads to reduced efficiency of apoptotic cell removal. Also, anti-C1q autoantibodies are seen in SLE patients and amplify local complement activation (Trouw et al., 2017).

### Similarities Between COVID-19 Infection and Autoimmune Diseases

There are some similarities in the pathogenesis and treatment of COVID-19 infection and autoimmune diseases. COVID-19 infection can result in cytokine storm with increased IL-1b, IL-2, IL-7, IL-8, IL-9, IL-10, IL-17, G-CSF, GM-CSF, IFN-γ, TNFα, IP10, MCP1, MIP1A, and MIP1B (Huang et al., 2020). In SLE and COVID-19 patients, increased IL-17 induces G-CSF resulting in kidney tissue damage. Also, IL-22 produced by Th17 cells is associated with SLE and COVID-19 pathogenesis by regulating antiapoptotic proteins, serum amyloid A (SAA) level, and fibrinogen production (Tse et al., 2004; Wu and Yang, 2020). Increased SAA is associated with higher COVID-19 severity and mortality, so anti-SSA drugs can be beneficial (Chen et al., 2020; Zinellu et al., 2021). Procoagulant changes and abnormal coagulation tests are seen in COVID-19 and SLE patients, which can be associated with the role of IL-6 in increasing fibrinogen levels (Hadid et al., 2021).

Also, Woodruff et al. focused on similarities between B-cell immunophenotypes in COVID-19 infection and SLE. They characterized a specific type of IgD and CD27 double-negative (DN) B cells in SLE that correlated the CXCR5-CD21^−^CD11c+ (DN2) group with disease severity (Woodruff et al., 2020). These B cells have extra-follicular that is consistent with the study of Kaneko et al., reporting the loss of germinal centers in the lymph nodes and spleens of acute COVID-19 patients as a result of irregular TNF production (Kaneko et al., 2020). In addition, the cell population is correlated with elevated inflammatory markers such as IL-6 and CRP and can produce autoantibodies (Woodruff et al., 2020).

ACE2, which converts angiotensin II to angiotensin-1-7, is a receptor of SARS-CoV-2 on epithelial cells (Rezaei et al., 2021). The binding of angiotensin II to the AT1 receptor triggers increased oxidative stress, inflammation, fibrosis, and vasoconstriction; however, angiotensin-1-7 stimulates vasodilation, antioxidant, and antiproliferative effects (Beyerstedt et al., 2021). Monteil et al. showed that human recombinant soluble ACE2 (hrsACE2) could protect from lung injury and prevent SARS-CoV-2 from entering the cell (Monteil et al., 2020). Inflammation in RA patients may lead to the involvement of endothelial cells and the development of atherosclerotic lesions, so lack of angiotensin II production by inhibiting ACE, an enzyme converting angiotensin I to angiotensin II, can improve vascular endothelial function (Sattar et al., 2003).

MS patients show elevated inflammatory cytokines such as IFN-γ, IL-12, TNF-α, and IL-17, along with the migration of Th1 and Th17 across the blood–brain barrier (Maxeiner et al., 2014). IL-6 and TGF-β stimulate STAT3 activation, leading to Th17 activation along with IL-17 and IL-21 secretion (Yang et al., 2007). MS patients have higher levels of osteopontin (OPN) in serum and cerebrospinal fluid (CSF). OPN is produced by T cells, B cells, macrophages, NK cells, dendritic cells, and neutrophils and acts as a pro-inflammatory cytokine by inducing the production of IL-12 in macrophages and IFN-γ in T cells and inhibition of IL-10 production in macrophages (Niino and Kikuchi, 2011). Also, increased IFN-γ and IL-12 lead to the upregulation of Th1 cells in the brain (Khaibullin et al., 2017). Th17 cells and IL-17 play an essential role in the pathogenesis of COVID-19 and MS (Dos Passos et al., 2016; Martonik et al., 2021).

Due to the similarities in pathogenesis, related therapeutic options can be proposed. For instance, fedratinib is a JAK2-specific inhibitor that inhibits Th17 cytokine production. Anifrolumab is an IFN-I signaling inhibitor that showed promising results in SLE patients. Also, chloroquine and hydroxychloroquine can bind to the virus and interfere with the glycosylation of the ACE2. Also, ACE inhibitors such as quinapril can inhibit TNF-α production and has an anti-inflammatory effect (Najafi et al., 2020).

### Autoimmune Bullous Dermatoses (AIBDs)

AIBDs include heterogeneous disorders which mainly consist of two groups of disorders: the subepidermal pemphigoid group which is named bullous and mucous membrane pemphigoid (MMP), and the intraepidermal pemphigus group which is named pemphigus vulgaris (PV) and foliaceus. These disorders have a common clinical feature, which is skin and mucosal blistering resulting in significant cutaneous damage accompanied by vast erosion formation (Drenovska et al., 2021). It is assumed that the underlying mechanisms in COVID-19, such as acute respiratory distress syndrome, extensive lung damage, and cytokine release storm, such as IL-1, IL-6, and TNF-α leading to interstitial pulmonary inflammation (Wang et al., 2020), could have impacts on these autoimmune disorders.

PV, as an autoimmune bullous disease affecting the mucosa and skin, is induced by autoantibodies against desmoglobin 1 and desmoglobin 3, which are adhesion proteins of the epidermis (Stanley and Amagai, 2006; Abdollahimajd et al., 2020). An approach for treatment of new cases of PV who are proven cases of COVID-19 has been suggested by Abdollahimajd et al. (Abdollahimajd et al., 2020). Intralesional or topical corticosteroid and dapsone should be considered in mild cases of PV (Abdollahimajd et al., 2020). IVIG as a therapeutic option could be administrated in severe cases of PV (Brown et al., 2018; Abdollahimajd et al., 2020). Not only may IVIG be the safest immunomodulatory for the long term in all age groups (Zhang and Liu, 2020) but it has also been suggested as a therapeutic option for COVID-19 (Wang et al., 2020). In cases with unavailability or unaffordability of IVIG, rituximab, which is a chimeric mouse/human anti–CD-20 monoclonal antibody, should be considered with patient monitoring and caution (Abdollahimajd et al., 2020).

Bullous pemphigoid (BP) is considered as subepidermal autoimmune disease, which is characterized by blister formation on an erythematous base and pruritis (Schmidt and Zillikens, 2013). Drenovska et al. reported a suspicious case of COVID-19 presented with newly diagnosed BP. He received a treatment course of systemic and topical corticosteroid which resulted in rapid control of BP (Drenovska et al., 2021). It should be considered that topical corticosteroids are safer than systemic therapy, particularly for extensive forms of BP (Joly et al., 2002). It has also been suggested to administer IVIG for parallel management of the conditions in BP-COVID-19 cases (Drenovska et al., 2021). In addition, doxycycline together with dapsone was effective in both COVID-19 and BP (Farouk and Salman, 2020).

### Myasthenia Gravis

Myasthenia gravis (MG) is one of the most common autoimmune disorders induced by autoantibody production against nicotinic acetylcholine receptors (AchRs) at the neuromuscular junction (Meriggioli and Sanders, 2009; Hübers et al., 2020). It has been proposed that an inflammatory reaction to a virus, as an external agent, can induce antibody production which also induces a triggered immune response. Cross-reaction of this immune response with the AchRs can happen due to molecular mimicry, which may lead to damage. It has been revealed that SARS-CoV-2 has affinity to ACE2 receptors, resulting in the autoantibody formation and an inflammatory cascade (Baig et al., 2020). These receptors are expressed in many organs, such as the kidneys, liver, and lungs. This will cause chemokine and proinflammatory cytokine production along with T and B cell depletion accompanied by high levels of TNF-α and interleukins which are associated with disease severity (Baig et al., 2020). Sriwastava et al. reported a positive case of COVID-19 who presented with left eye diplopia and fatigable ptosis. A combination of laboratory investigations, findings from history, and electrodiagnostic testing confirmed the diagnosis of MG. She received a course treatment of 60 mg of pyrodostigmine every 6 hours, which was followed by subjective improvement of her ptosis and diplopia (Sriwastava et al., 2021a).

### Hashimoto’s Thyroiditis

Hashimoto’s thyroiditis, characterized by thyroid-specific autoantibodies, is considered as one of the most common autoimmune disorders (Ralli et al., 2020). Tee et al. reported a case of COVID-19, without any personal or family history of thyroid or autoimmune disease, who complained of muscle weakness and severe acute-onset generalized fatigue after resolution of the respiratory symptoms. Laboratory findings confirmed the diagnosis of Hashimoto’s thyroiditis. After receiving levothyroxine 25 mcg/day for 5 weeks, his symptoms have been resolved (Tee et al., 2020).

### Systemic Lupus Erythematosus (SLE)

SLE is a member of autoimmune diseases associated with production of pathogenic autoantibodies and involvement of multiple organs (Zamani et al., 2021).

Several studies reported SLE following SARS-CoV-2 infection. For example, Zamani et al. reported a case of a patient who developed SLE, 2 months after SARS-CoV-2 infection (Zamani et al., 2021). Therapy in the patient was with methylprednisolone (1,000 mg for three consecutive days), hydroxychloroquine and prednisolone (30 mg daily), IV cyclophosphamide (1,000 mg monthly), gabapentin, and vitamin B (300 mg daily), which significantly improved the health status of the patient. At first, treatment with methylprednisolone pulse (1,000 mg) was performed for three consecutive days, and then hydroxychloroquine and prednisolone (30 mg per day) were prescribed to the patient. As a result of this treatment, platelets were reduced to 100,000/mm3 and hemoglobin to 11 g/dl, but paresthesia, proteinuria, and edema persisted. The patient also received monthly doses of 1,000 mg of intravenous cyclophosphamide. The patient also received monthly doses of 1,000 mg of intravenous cyclophosphamide. The patient was discharged but was nevertheless receiving hydroxychloroquine, prednisolone (10 mg dai-ly), cyclophosphamide, gabapentin, and B vitamins (300 mg daily). The patient was followed up with after 6 months. The results were as follows: paresthesia was enhanced. Laboratory tests (CBC, ESR, CRP, T3, and T4) were normal, and urine protein was 230 mg/day. The double-stranded anti-DNA antibody was decreased to the normal range (<35 IU/ml) (Zamani et al., 2021). Also, Bonometti et al. reported a case with SLE following SARS-CoV-2 infection in which treatment with piperacilline/tazobactam, steroid therapy, hydroxychloroquine, and oxygen supplementation was successfully achieved (Bonometti et al., 2020).

One possible explanation for this condition is that infection with SARS-CoV-2 causes severe immune activation, cytokine storm (upregulation in tumor necrosis factor, interferon gamma, IL-2, and other cytokines), thus indicating a form of MAS. Moreover, patients with SLE can develop cytokine storm (elevation of cytokines including TNF-α, IFN-γ, IL-1, IL-6, and IL-18) and MAS more easily (Spihlman et al., 2020).

### Systemic Lupus Erythematosus and Varicella‐Like Rash

Slimani et al. reported a case with no previous medical history, who was infected with SARS-CoV-2 and was diagnosed with SLE and APS and developed a COVID‐19–related varicella-like rash on the trunk. The treatment included steroid therapy with methylprednisolone and a single dose of chloroquine (Slimani et al., 2021). It is hypothesized that SARS-CoV-2 infection can cause autoimmunity. Previous studies reported that viruses could cause autoimmunity by mechanisms such as molecular mimicry, epitope spreading, immortalization in infected B cells, and bystander activation. Furthermore, SARS-CoV-2 infection increases the release of various cytokines, causing a disorder in acquired and innate immune response, which might contribute to the condition. Also, it has been reported that skin symptoms are secondary immune responses to nucleotides of the virus (Slimani et al., 2021).

### Graves’ Disease

Grave’s disease is an organ-specific autoimmune disorder in which the binding of the autoantibodies to the thyroid-stimulating hormone receptor (TSHR) increases thyroid function leading to hyperthyroidism (Davies et al., 2020).

Salat et al. reported two cases of patients who developed Grave’s disease following SARS-CoV-2 infection. One of the patients had a previous history of Graves’ disease and has been in remission during the past 35 years, and the other had no history of thyroid disease. Both patients showed suppressed levels of TSH and were positive for TSH receptor, thyroperoxidase, and thyroglobulin antibodies. Treatment was started with thiamazole and propranolol in both patients and resulted in improvement of thyroid function and complications (Mateu-Salat et al., 2020).

It has been suggested that the hyper-inflammation caused by severe SARS-CoV-2 infection can trigger the development of Grave’s disease. SARS-CoV-2 infection leads to increased IL-6 and Th1 cytokines, whereas autoimmune response in Graves’ disease is mainly associated with Th2 cells. However, IL-6 upregulation is also seen in Grave’s disease. IL-6 can exert various complex functions by interacting with cellular receptors (Jiménez-Blanco et al., 2021). Also, IL-6 can inhibit Th1 polarization and promote the Th2 response through stimulation of IL-4 secretion and inhibition of IFN-γ secretion by CD4 T cells (Velazquez-Salinas et al., 2019).

### Generalized Pustular Psoriasis

Generalized pustular psoriasis is a scarce demonstration of psoriasis (chronic inflammatory disease) which could be provoked by drugs, viral infections, pregnancy, and variety of medications. Dadras et al. reported a patient with history of psoriasis in childhood who developed generalized pustular psoriasis after COVID-19 infection (Shahidi Dadras et al., 2021).

A hyperinflamatory state in SARS-CoV-2 infection can cause psoriasis manifestation. Also, hydroxychloroquine, a member of important drugs for COVID-19 therapy, can cause psoriasis or lead to lesion recurrence or exacerbation. The condition occurs because hydroxychloroquine influences cholesterol metabolism, which is essential for the skin to function as a barrier, thus leading to weakness of the surface layer of the skin and abnormal keratinocyte proliferation. Treatment for psoriasis in the patient was with systemic retinoids (Shahidi Dadras et al., 2021).

### Guillain-Barre Syndrome (GBS)

GBS is a multi-form immune-mediated polyradiculoneuropathy recognized by both sensory and motor symptoms depending on the disease subtype. The most common subtype of GBS, acute inflammatory demyelinating polyradiculoneuropathy (AIDP), is typically characterized by progressive muscle weakness leading to paralysis and sensory deficits (Van den Berg et al., 2014; Willison et al., 2016). The majority of GBS incidences are due to a pre-existing infection such as cytomegalovirus (CMV), Epstein–Barr virus (EBV), Zika virus, influenza virus, and *Campylobacter* jejuni (Jacobs et al., 1998; Cao-Lormeau et al., 2016). The process of post-infection GBS remains to be fully understood; however, in the case of *Campylobacter* jejuni, it is established that the cross-reaction of host antibodies (produced against bacterial antigen) with human peripheral nerve results in neural damage (Yuki and Hartung, 2012). It is possible that this event might be true for other GBS-related pathogens in which there is a structural resemblance between foreign antigen and host nerve glycolipids since anti-ganglioside antibodies are detected in almost 60% of GBS patients (Kaida et al., 2009). In January 2020, the very first report of a patient with co-existing COVID infection and GBS was published. A 61-year-old female was admitted to the hospital with acute muscle weakness and laboratory results confirming GBS. Later, she developed COVID-19 symptoms on day 8 of GBS. Given her history of visiting Wuhan a week prior to admission and primary laboratory results (lymphocytopenia and thrombocytopenia), an asymptomatic COVID-19 infection on admission was assumed (Zhao et al., 2020). This was followed by the various case-series reports indicating a presumable relation between COVID-19 infection and GBS (Caress et al., 2020; Sriwastava et al., 2021b). To date, there is no absolute explanation for this possible link, while some theories have been proposed. The same resemblance theory is suggested; however, no homology between peripheral nerve tissue and SARS-CoV-2 has been discovered yet. Also, the detection of anti-ganglioside antibodies was uncommon in the majority of reports on COVID-associated GBS (Caress et al., 2020; Sriwastava et al., 2021b). Alternatively, some authors speculated that nerve damage may also set in due to T cell activation and cytokines released from macrophages in response to SARS-CoV-2 (Hartung and Toyka, 1990). Nevertheless, the population-based data in the United Kingdom failed to show a temporal relationship between GBS and COVID. Keddie et al. (Keddie et al., 2021) hypothesized that the lockdown policy during the pandemic and more cautious behavior may play a role in reducing the transmission of other GBS-related pathogens. In this scenario, the increase of GBS incidence in COVID-19 patients is the indirect result of a decrease in GBS cases caused by other pathogens.

### Autoimmune Disease Following Vaccination

#### Autoimmune Hepatitis

Autoimmune hepatitis is known as a form of chronic hepatitis with an unknown cause (Krawitt, 2006). Clayton-Chubb et al. reported a case of COVID-19 vaccine–related liver injury. The patient presented with autoimmune hepatitis 26 days after the first dose of the vaccine (Oxford-AstraZeneca) injection. He received a treatment course of prednisolone 60 mg/day. After a few weeks, his general condition improved and the dosage was tapered to 20 mg/day (Bril et al., 2021). This was the first case report of autoimmune hepatitis following COVID-19 vaccination. Further investigation is still required to determine whether there is a causal relationship. Also, other factors such as drugs or toxins may contribute to the condition and their role should be considered in future studies.

### Long COVID

Some COVID-19 patients, from mild to severe forms of the disease, may present with debilitating and variable symptoms for several months after the initial diagnosis of COVID-19. This condition, which is called “Long COVID,” typically refers to the symptoms lasting for 2 months or longer subsequent to infection (Ortona et al., 2021). The virus may activate an excessive inflammatory response resulting in damage of organs. In addition to this mechanism, an autoimmune reaction which is unmasked by SARS-CoV-2 may have role in Long COVID’s symptoms. The higher incidence of long COVID in females can be justified by the autoimmune hypothesis. In order to identify specific and personalized treatments for this syndrome, it is important to study the appearance and the characterization of autoantibodies in the serum of patients (Ortona et al., 2021).

## Conclusion

As investigated in this study, there could be an association between COVID-19 disease and autoimmune diseases as well as a multisystem inflammatory syndrome. There are similarities in the immune responses to both diseases, and it should be stated that the damage in both diseases occur to a large extent due to the malfunction of the immune system. Although the main target of SARS-CoV-2 is the lungs, it should be noted that it can affect the function of other organs. Although the exact mechanism of post-COVID-19 autoimmune disease development is unclear, some factors such as pro-inflammatory cytokines and chemokines, damage-associated molecular patterns (DAMPs), molecular mimicry, cross-reactive antibodies, and auto-antibodies were hypothesized to attribute to the diseases. Reports indicated that the spectra of autoimmune and autoinflammatory conditions in SARS-CoV-2–infected populations are mostly responsive to IVIG therapy. Early diagnosis of COVID-19–linked autoimmune and autoinflammatory diseases and prompt initiation of therapy are crucial for successful recovery and preventing end-organ damage and fatality. MIS has also followed the footsteps of the COVID-19 and has been presented as a rare, but life-threatening, complication of the disease, especially in children. Efforts to minimize the risk of exposure to COVID-19 in children, especially those from socioeconomically disadvantaged populations, and prompt recognition of the syndrome, are keys to limit the incidence of this febrile syndrome.
